# Head-to-head comparisons of breast density assessment models using deep learning on digital and synthetic mammograms

**DOI:** 10.1117/1.JMI.13.2.024503

**Published:** 2026-04-22

**Authors:** Krisha Anant, Juanita Hernández López, Junjie Cui, Sneha Das Gupta, Debbie L. Bennett, Aimilia Gastounioti

**Affiliations:** a Washington University School of Medicine, Computational Imaging Research Center, Breast Image Computing Lab, St Louis, Missouri, United States; b Washington University School of Medicine, Department of Radiology, St Louis, Missouri, United States; c Barnes-Jewish Hospital, Washington University of School of Medicine, Siteman Cancer Center, St Louis, Missouri, United States

**Keywords:** breast density, breast imaging reporting and data system, deep learning, artificial intelligence, digital mammogram, synthetic mammogram, digital breast tomosynthesis, breast cancer risk, breast cancer screening

## Abstract

**Purpose:**

We aim to evaluate the performance of different deep learning (DL) architectures in breast density classification using digital mammograms (DMs) and synthetic mammograms (SMs) from digital breast tomosynthesis (DBT).

**Approach:**

We retrospectively analyzed routine mammographic screening exams (Selenia Dimensions, Hologic Inc.) acquired between 2015 and 2018 at our institution. Each mammogram dataset (DM and SM) included 10,000 exams representing all four breast imaging reporting and data system density categories (a to d). We used ResNet-50, EfficientNet-B0, and DenseNet-121 architectures, separately fine-tuned for breast density classification with DM and SM. Classification accuracy was assessed on 10% unseen test sets in four-category (a to d) and binary (nondense versus dense) scenarios. Evaluations also considered mammogram view (craniocaudal [CC] versus mediolateral-oblique [MLO] view) and race (White versus Black women).

**Results:**

DL architectures showed detectable, yet small, differences in classification accuracy regardless of mammogram format. ResNet-50 achieved a four-category accuracy of 0.727 (95% CI: [0.713, 0.740]) for DM, higher than 0.713 (95% CI: [0.699, 0.728]) for SM (p=0.151). EfficientNet-B0 and DenseNet-121 showed similar trends. DM-SM differences for binary classification were of similar magnitude but statistically significant (p<0.05), with test accuracies ranging from 0.871 to 0.920. The MLO view generally outperformed the CC view, and the results were consistent across racial groups.

**Conclusions:**

We highlight that various DL architectures perform effectively in breast density classification, emphasizing the significance of mammogram format and view, though results may vary with different vendors. These insights are crucial for enhancing DL-based breast density assessment, especially during the shift from DM to DBT.

## 1 Introduction

Breast density refers to the amount of fibroglandular or “dense” tissue within the breast. Breast density, typically assessed through mammography, is an important biomarker in breast cancer screening because dense breast tissue increases the risk of breast cancer^1^ and may also impair breast cancer detection on mammography.^2^ The most used breast density assessment method in the clinical setting is visual grading, wherein breast density is classified by interpreting radiologists based on the American College of Radiology (ACR) breast imaging reporting and data system (BI-RADS).^3^ The BI-RADS breast density classification includes four categories of increasing breast density (a, almost entirely fatty; b, scattered areas of fibroglandular density; c, heterogeneously dense, which may obscure small masses; and, d, extremely dense, which lowers the sensitivity of mammography), which are associated with increasing levels of breast cancer risk.^4^ However, BI-RADS breast density assessment often is limited by inter-radiologist variability, leading to inconsistent results.^5^^–^^7^

To enhance robustness in breast density assessment, various deep learning (DL) models have been developed to automate BI-RADS breast density classification with mammography, demonstrating also potential for improved longitudinal consistency in breast density evaluation in clinical settings.^8^^,^^9^ These DL models have demonstrated moderate to strong performance, achieving four-category density classification accuracies in the range of 67% to 90%.^9^^–^^13^ Most models have been based on pre-trained architectures of residual neural networks (ResNets),^14^ fine-tuned for 2D full-field digital mammograms (DMs). For example, using ResNets as backbone architectures in DL, Khara et al.^13^ achieved a four-category density classification accuracy of 0.81, whereas Chang et al. reported an accuracy of 0.67,^11^ which are representative of the higher and lower ends of DL-based density classification performance achieved to date. The architectures of dense convolutional networks (DenseNets)^15^ and efficient convolutional networks (EfficientNets)^16^ have also been commonly used as backbone architectures in various density DL models for mammography.^9^^,^^17^

Despite these efforts and the promising results that they have generated, head-to-head comparisons of different DL architectures across studies are missing due to differing datasets, mammographic imaging protocols, and DL model configurations. As a result, prior work has not systematically compared accuracy across DL architectures, which could help identify potential effects of DL model design in breast density assessment. In addition, the use of 2D synthetic mammogram (SM),^18^ a newer 2D mammogram format obtained with digital breast tomosynthesis (DBT),^19^^,^^20^ remains largely unexplored in DL models for breast density classification.^21^^,^^22^ However, studies have compared visual breast density gradings by radiologists on DM versus SM images, finding clinically significant effects of different mammogram formats.^23^ Prior studies have also shown that the mammogram format may also impact conventional quantitative imaging metrics extracted from mammograms, such as texture, radiomics, and continuous non-DL-based measures of breast density.^24^^,^^25^ Because SM images have somewhat different appearances than DM images (owing to differences in image processing) and because DM images may soon no longer be available for breast density assessment in DBT imaging, it is important to understand the potential impact of DM versus SM on the assessment of breast density with DL.

Our study addresses these two important gaps by performing head-to-head comparisons of DL models for BI-RADS breast density classification across widely used backbone architectures and both 2D mammogram formats (DM and SM). Building on Anant et al.,^26^ this study uses a significantly expanded dataset of Hologic mammograms, enhanced image segmentation and quality control methods, and extensive subgroup evaluations to provide valuable insights into the influence of mammogram format, view type, and backbone DL architecture on breast density classification accuracy, using uniformly curated datasets and standardized preprocessing steps. The goal is to identify optimal strategies for automated DL-driven breast density assessment that may enhance breast cancer risk stratification and screening practices.

## 2 Methods

### 2.1 Study Dataset

**Fig. 1:**
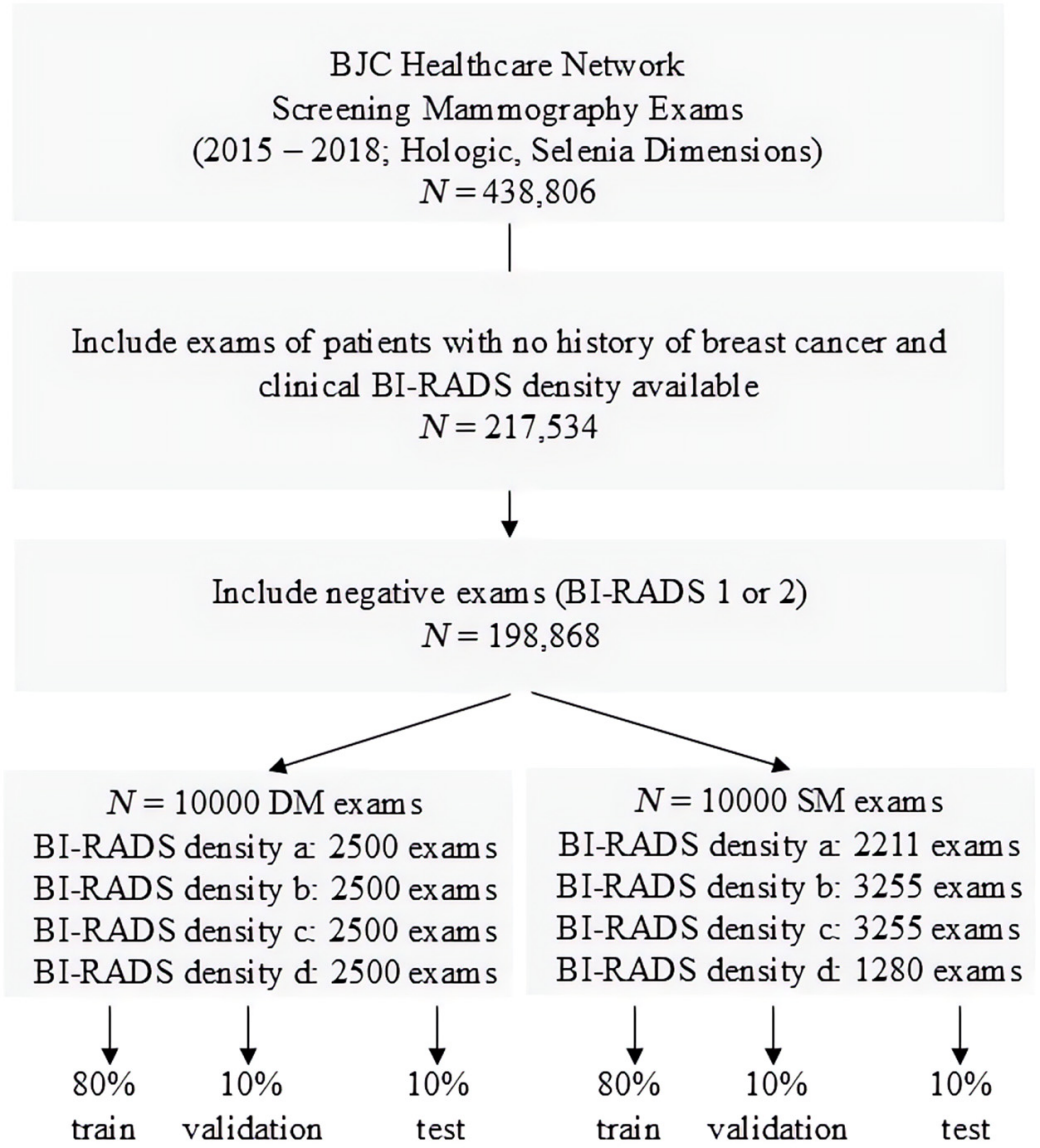
Diagram of data curation. Mammography screening data were acquired from a large breast cancer screening practice, filtered for clinical BI-RADS density availability and breast cancer history, and separated into digital mammogram (DM) and synthetic mammogram (SM) datasets for training and testing of deep learning models.

In this institutional review board–approved, Health Insurance Portability and Protection Act–compliant study under a waiver of consent (IRB ID: 202203188; WashU Human Research Protection Office), we retrospectively analyzed BI-RADS 1 and 2 routine mammographic screening exams (Selenia Dimensions, Hologic Inc.) acquired from patients with no history of breast cancer within the Barnes-Jewish/Christian (BJC) Healthcare network in St. Louis, MO, between 2015 and 2018. For each 2D mammogram format—DM and SM—we curated datasets of 10,000 exams with enhanced representation from all four BI-RADS density categories (Fig. 1). Each exam included at least one mediolateral oblique (MLO) and one craniocaudal (CC) view per breast of a woman. Reference BI-RADS breast density assessments and race information for all exams were retrieved from the BJC mammography reporting software (Magview 7.1).

Each dataset was split into training, validation, and test sets with 80%, 10%, and 10% of the data, respectively, while ensuring consistent distribution of BI-RADS density categories across data splits and maintaining that all images from the same exam were kept within the same data split. Exams from the same patient were also assigned to the same training-validation-test data split to prevent data leakage.

**Fig. 2:**
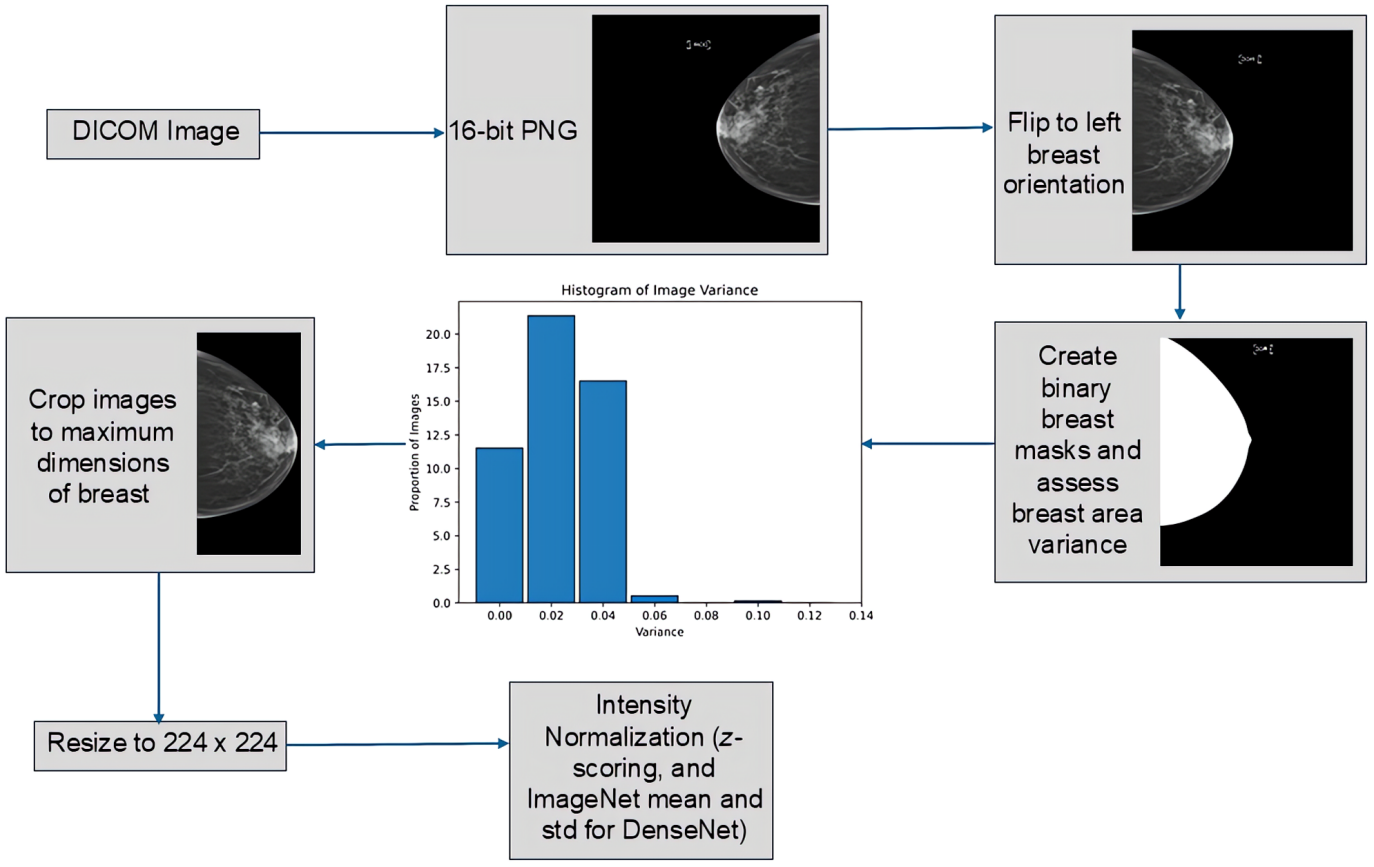
Flowchart showing image pre-processing steps. DICOM images were converted to a PNG file format and were, then orientation-, size-, and intensity-standardized. A quality control step to exclude potential breast segmentation artifacts was also applied.

All images, initially in DICOM format, were converted to 16-bit grayscale PNGs, flipped into left breast orientation, and used to create binary breast masks with previously validated breast segmentation methods.^27^^,^^28^ that detect the boundary of the breast tissue with the image background and the pectoralis muscle (Fig. 2). For each exam, we used the binary masks to assess the variance in breast area across all views available for the exam; this quality-control step assumes that low variance in breast area over mammogram views is overall expected due to breast bilateral symmetry and large variance is likely a sign of problematic breast segmentations.^29^^,^^30^ Most images were within a variance of 0.05, with a maximum of 0.12 variance, so no exams were excluded. The images were then cropped to the maximum dimensions of the breast in the mask. The resulting cropped images were resized to 224×224  pixels, based on multiple studies in mammography and breast density classification that successfully employed this resolution.^9^ Image intensity was normalized via z-scoring for all DL architectures, except for DenseNet that expects input pixel values to be scaled to the [0, 1] range and then normalized with the mean and standard deviation values from the ImageNet dataset.^15^

### 2.2 Model Architectures and Hyperparameter Optimization

We implemented the DL architectures of ResNet-50, EfficientNet-B0, and DenseNet-121 with initial weights adapted from ImageNet. Briefly, ResNet-50 utilizes skip connections to train deeper networks,^14^ and EfficientNet-B0 employs a compound scaling method to balance model size and computational efficiency.^16^ DenseNet-121 is a convolutional neural network architecture that uses dense connections, where each layer receives input from all preceding layers, allowing for more efficient gradient flow and feature reuse, which reduces the number of parameters and helps alleviate the vanishing gradient problem.^15^ All models incorporated convolutional layers, a global average pooling (GAP) layer, and a fully connected layer for classification and were fine-tuned for four-category breast density classification, separately for DM and SM, via a two-step training strategy. The top classification layers were first trained with a higher learning rate. whereas the pretrained backbone was frozen, and then, backbone layers were unfrozen and fine-tuned at a much lower learning rate to adapt the pretrained features. Training also included image augmentation (rotation, translation, flipping, and contrast) to enhance generalization. Hyperparameter optimization was performed considering batch sizes of 32, 64, and 128, learning rates of ${1e}^{-4}$, ${1e}^{-5}$, and ${1e}^{-6}$, and two optimizers (Adam and RMSprop).

### 2.3 Performance Evaluation

For each mammogram format and DL architecture, we assessed breast density classification accuracy on the unseen test sets, in four-category (BI-RADS density a to d) and binary (nondense: a to b; dense: c to d) breast density classification scenarios, with 95% confidence intervals estimated via 1000 bootstrapping iterations. We also used confusion matrices to assess the predicted versus reference density categories. Evaluations were performed across all views and separately in CC and MLO views. For assessments with all views, each model was trained on both CC and MLO views but made a prediction for each view independently; the reported performance is therefore the aggregate performance across this entire set of single-view predictions. In addition, to assess potential effects of race on density DL performance, we evaluated the best-performing DL architecture in both mammogram formats by race. Comparisons among DL architectures, mammogram formats, views, and race were performed via permutation tests.

## 3 Results

Our study dataset comprised 10,000 DM and 10,000 SM exams (Table 1). Compared with the DM set, the SM set included women of slightly higher mean age at screening (58.4±10.7 versus 56.9±10.4 years) and a higher proportion of Black women (34.9% versus 27.9%).

**Table 1:** Study dataset characteristics.

	DM	SM
Age (years)	56.9 ± 10.4	58.4 ± 10.7
Race		
White	5953 (59.5%)	6073 (60.7%)
Black	2794 (27.9%)	3486 (34.9%)
Other/unknown	1253 (12.5%)	441 (4.4%)
Reference BI-RADS density		
a	2500 (25.0%)	2211 (22.1%)
b	2500 (25.0%)	3255 (32.6%)
c	2500 (25.0%)	3255 (32.6%)
d	2500 (25.0%)	1280 (12.7%)

Data are means ± standard deviations for continuous variables, and numbers of patients with percentages in parentheses for categorical variables. p-value indicates the difference between DM and SM via the Wilcoxon rank sum test for continuous variables and the chi-square test for categorical variables.

Optimal hyperparameters for ResNet-50 with SM and DM images and EfficientNet-B0 with SM images were found to be a batch size of 64, a learning rate of ${1e}^{-6}$, and the Adam optimizer. Optimal hyperparameters for EfficientNet-B0 with DM and DenseNet-121 with SM and DM were a batch size of 32, a learning rate of $1e^{-6}$, and the Adam optimizer.

Using these optimal hyperparameters, ResNet-50 and DenseNet-121 generally achieved slightly higher four-category classification accuracy on DM and SM, whereas differences in binary classification were more substantial (Table 2). ResNet-50 achieved a test accuracy of 0.727 (95% CI: [0.713, 0.740]) in four-category density classification for DM, which was slightly higher than its corresponding test accuracy of 0.713 (95% CI: [0.699, 0.728]) in SM (p=0.151). Similar differences between DM and SM were also found for EfficientNet-B0 and DenseNet-121. Differences between DM and SM were of similar magnitude but significant (p<0.05) for binary breast density evaluations for all DL architectures, with test accuracies ranging from 0.871 to 0.920. When mammogram views were compared, the MLO view generally performed better than the CC view. To assess the potential effect of class imbalance on our results, we also conducted a sensitivity analysis on DM and SM test sets with 128 exams per density class (to match the number of category d exams in the test set of SM). Although the magnitude of differences in binary density classification was attenuated under the balanced setting, the overall conclusions remained unchanged (Appendix Table 4).

**Table 2:** Breast density classification performance on the test set of each evaluation scenario.

	4-category density classification			Binary density classification		
	DM	SM	$p_{1}$	DM	SM	$p_{1}$
	ResNet-50 architecture					
All views	0.727 [0.713, 0.740]	0.713 [0.699, 0.728]	0.151	0.915 [0.907, 0.924]	0.890 [0.881, 0.900]	<0.001
CC views	0.723 [0.705, 0.743]	0.710 [0.691, 0.729]	0.329	0.912 [0.899, 0.923]	0.892 [0.878, 0.904]	0.028
MLO views	0.730 [0.712, 0.748]	0.716 [0.697, 0.735]	0.302	0.919 [0.907, 0.929]	0.890 [0.876, 0.903]	0.002
	EfficientNet-B0 architecture					
All views	0.700 [0.686, 0.714]	0.698 [0.684, 0.711]	0.810	0.911 [0.902, 0.919]	0.881 [0.871, 0.891]	<0.001
CC views	0.700 [0.681, 0.721]	0.682 [0.663, 0.702]	0.205	0.903 [0.890, 0.916]	0.871 [0.857, 0.885]	0.001
MLO views	0.700 [0.681, 0.717]	0.713 [0.693, 0.732]	0.368	0.918 [0.906, 0.928]	0.891 [0.877, 0.904]	0.002
	DenseNet-121 architecture					
All views	0.733 [0.718, 0.746]	0.711 [0.698, 0.725]	0.029	0.920 [0.912, 0.928]	0.885 [0.875, 0.895]	<0.001
CC views	0.732 [0.714, 0.750]	0.713 [0.694, 0.733]	0.200	0.920 [0.909, 0.932]	0.886 [0.872, 0.900]	<0.001
MLO views	0.734 [0.716, 0.752]	0.709 [0.691, 0.728]	0.076	0.920 [0.909, 0.930]	0.884 [0.871, 0.898]	<0.001

Breast density classification accuracy on the test set. Numbers in parentheses are 95% CIs.

$p_{1}$: p-value for the difference in accuracy between DM and SM using a permutation test.

**Fig. 3:**
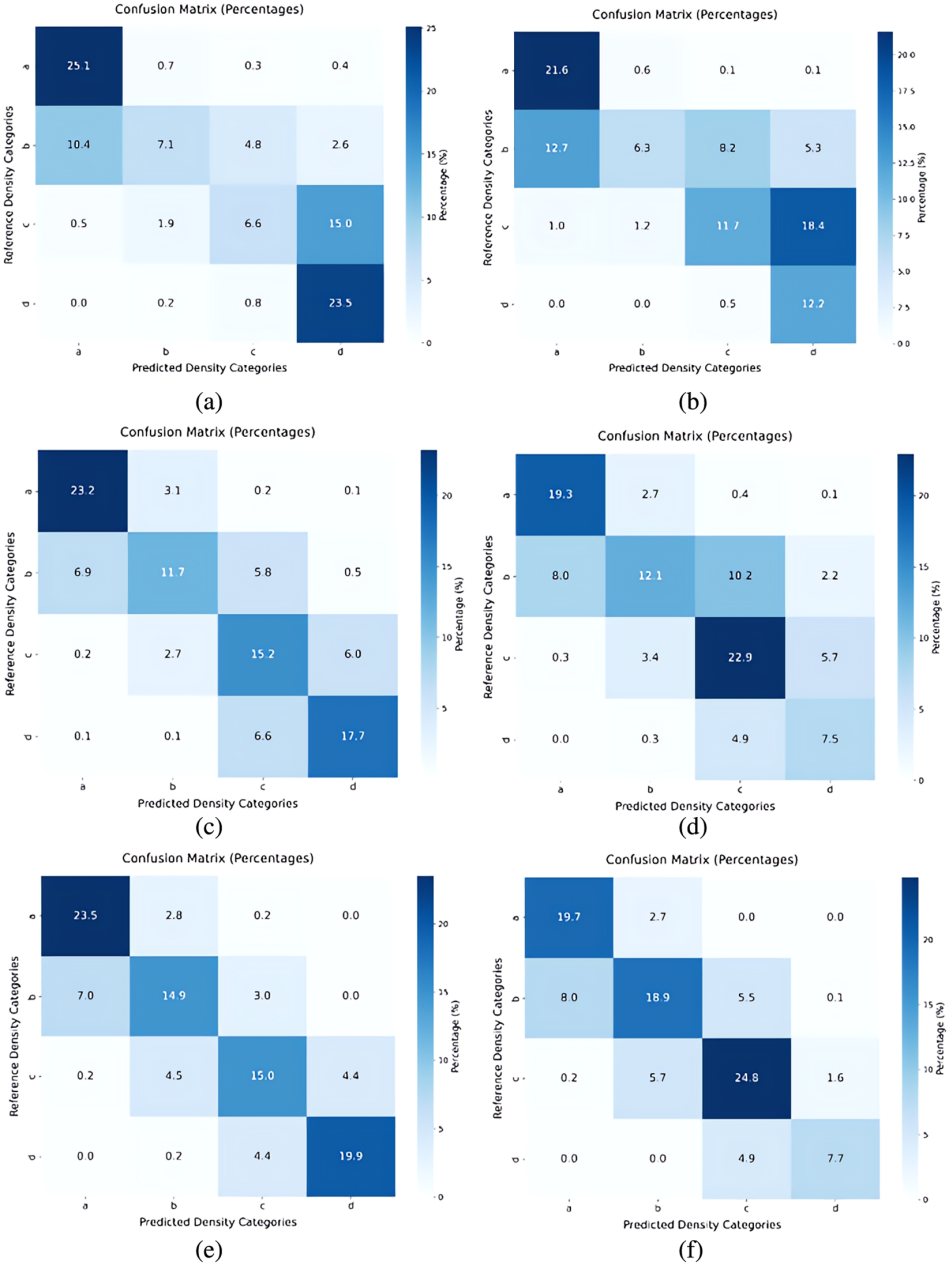
Confusion matrices showing density classification accuracies of (a) DM with ResNet-50, (b) SM with ResNet-50, (c) DM with EfficientNet-B0, (d) SM with EfficientNet-B0, (e) DM with DenseNet-121, and (f) SM with DenseNet-121.

There were also differences in classification accuracy by breast density category (Fig. 3). DM exams in breast density categories a and d were predicted the most accurately; classification accuracy for DM exams with reference density categories b and c was less accurate. In SM exams, the lowest classification accuracy was observed in density category d likely because of the lower representation of density category d in the SM dataset (Table 1). In both mammogram formats, there tended to be more confusion between adjacent density categories and confusion between nondense and dense categories was generally less common than confusion within the category.

When evaluations were stratified by race (Table 3), breast density classification performance was comparable both in DM (e.g., for ResNet-50, White women: 0.718 versus Black women: 0.719) and in SM (e.g., for ResNet-50, White women: 0.708 versus Black women: 0.723). Moreover, similar performance differences between DM and SM persisted in White and Black women, which were significant in the context of binary density classification.

**Table 3:** Breast density classification performance of all models on the test set, overall and separately for Black and White women.

	4-category density classification			Binary density classification		
	DM	SM	$p_{1}$	DM	SM	$p_{1}$
	ResNet-50 architecture					
All women	0.727 [0.713, 0.740]	0.713 [0.699, 0.728]	0.151	0.915 [0.907, 0.924]	0.890 [0.881, 0.900]	<0.001
White	0.718 [0.701, 0.736]	0.708 [0.690, 0.727]	0.441	0.908 [0.895, 0.919]	0.882 [0.867, 0.894]	<0.001
Black	0.719 [0.694, 0.743]	0.723 [0.697, 0.746]	0.859	0.920 [0.904, 0.935]	0.902 [0.885, 0.916]	0.128
	EfficientNet-B0 architecture					
All women	0.700 [0.686, 0.714]	0.698 [0.684, 0.711]	0.810	0.911 [0.902, 0.919]	0.881 [0.871, 0.891]	<0.001
White	0.688 [0.671, 0.705]	0.688 [0.670, 0.708]	0.999	0.898 [0.886, 0.909]	0.873 [0.866, 0.886]	0.005
Black	0.692 [0.667, 0.718]	0.725 [0.702, 0.748]	0.069	0.920 [0.904, 0.936]	0.901 [0.883, 0.917]	0.088
	DenseNet-121 architecture					
All women	0.733 [0.718, 0.746]	0.711 [0.698, 0.725]	0.029	0.920 [0.912, 0.928]	0.885 [0.875, 0.895]	<0.001
White	0.728 [0.708, 0.745]	0.708 [0.690, 0.725]	0.108	0.913 [0.902, 0.924]	0.875 [0.863, 0.888]	<0.001
Black	0.713 [0.688, 0.737]	0.716 [0.690, 0.740]	0.893	0.926 [0.912, 0.940]	0.900 [0.883, 0.916]	0.019

Breast density classification accuracy on the test set. Numbers in parentheses are 95% CIs. p-value: for the difference in accuracy between DM and SM.

## 4 Discussion

In this study, we evaluated the performance of three widely used DL architectures—ResNet-50, EfficientNet-B0, and DenseNet-121—for the task of breast density classification using two formats of 2D mammograms: conventional DM and SM acquired with DBT. Our findings indicate that there are detectable, yet small, classification performance differences among the models when trained with similar sample sizes and training strategy, irrespective of the mammogram format. We also observed that DM outperformed SM, and CC views generally outperformed MLO views in both mammogram formats. Importantly, these conclusions held consistently for both Black and White women, demonstrating the generalizability of our findings across racially diverse mammogram datasets. Our findings can inform the development of breast density DL models toward optimizing their performance, particularly in the transition of mammographic screening from DM to DBT.

Different DL architectures employ distinct arrangements of layers, filter sizes, and connection patterns. This leads to them learning different sets of features or patterns from the input image. For example, one architecture might be more sensitive to edges and textures, whereas another might focus more on global shapes or intensity distributions in its early layers. While employing diverse internal mechanisms for feature extraction, DL architectures ultimately converge on aggregating information relevant to the classification task. Our findings suggest that, although the path to feature extraction may differ, the final aggregated information crucial for accurate classification of a mammogram into distinct density categories tends to converge on similar underlying semantic content. This allows different DL architectures to achieve comparable performance in the task of breast density classification with mammography, despite their internal variations.

DBT is the latest evolution of mammography, rapidly replacing conventional DM in the United States. Prior research comparing the visual breast density assessments by radiologists on DM and SM images has identified clinically significant differences attributable to the mammogram format.^23^ Furthermore, prior studies have demonstrated that the mammogram format can influence conventional quantitative imaging metrics derived from mammograms, including texture analysis, radiomics, and continuous non-DL-based measures of breast density.^24^^,^^25^ Our study showed that these differences may also influence the performance of DL models of BI-RADS breast density. However, it remains unknown whether a single DL model for breast density classification could support both mammogram formats effectively.

There are various reasons why MLO views might have performed differently from CC views in DL-driven breast density classification. First, the CC view provides a top-down perspective of the breast, which typically includes a more uniform distribution of tissue and less overlapping structures compared with the MLO view. Conversely, oblique angles in MLO views can introduce greater variability. In addition, although the clinical BI-RADS breast density assessment is a qualitative process designed as a visual observation of both the MLO and CC views by the interpreting radiologists, studies have hypothesized that radiologists may “subconsciously” use only one view type in assigning a BI-RADS breast density category.^31^ Our findings of higher classification performance in CC view reinforce this hypothesis, suggesting that potentially it is the MLO view that radiologists may have predominantly used to determine the breast density BI-RADS categories used as reference in our analysis.

Our study has several notable strengths. The standardized sample size for both mammogram formats—DM and SM—facilitates fair and accurate comparisons, allowing us to draw meaningful conclusions about the performance of different DL architectures across various mammogram formats. In addition, by including both conventional DM and SM acquired with DBT, we address the latest advancements in mammography and provide insights that are highly relevant for current and future clinical practices. Moreover, the employment of multiple widely used DL architectures underscores the comprehensiveness and robustness of our approach. Evaluating these varied architectures ensures that our conclusions are not architecture-specific but applicable broadly across different DL models. Last, our findings are consistent across racially diverse mammogram data, with results demonstrating robustness in both Black and White women. This generalizability is vital as it highlights the potential of our findings to be applied across different demographic groups.

There are also limitations to our study. First, our data originate from a single site, and we did not perform external validation of our results. As this study was a technical investigation primarily focused on comparing DL architectures and mammogram formats, our models were not optimized to achieve the highest possible accuracies. Second, the study exclusively used mammograms from one vendor (Hologic), which may limit the generalizability of our findings. Mammograms can vary significantly between vendors, and even within the same vendor, SM formats have evolved—for instance, “C-View” and “Intelligent 2D” are different SM formats from Hologic. These variations could influence the performance of DL models. Last, breast density categories were less balanced in the SM dataset compared with the DM dataset. Although our sensitivity analysis results with balanced test sets were consistent with our main conclusions, more balanced training sets for SM could offer additional insights into comparisons between DM and SM. Our future research steps will address these limitations by including data from different sites and vendors, as well as incorporating quantitative measures of breast density^32^^–^^36^ to enhance the robustness and applicability of our findings.

## 5 Appendix: Sensitivity Analysis of Breast Density Classification Performance Across Imaging Modalities and Model Architectures

Table 4 presents a sensitivity analysis comparing breast density classification accuracy between DM and SM across ResNet-50, EfficientNet-B0, and DenseNet-121 architectures, stratified by view (all, CC, and MLO) for both four-category and binary classification tasks. Overall, performance is similar between DM and SM (permutation test $p_{1}$ > 0.05 for most comparisons), with statistically significant differences observed in binary classification for DenseNet-121.

**Table 4:** Sensitivity analysis: Breast density classification performance on subsampled DM and SM test sets with matched representations from each BI-RADS density category.

	4-category density classification			Binary density classification		
	DM	SM	$p_{1}$	DM	SM	$p_{1}$
	ResNet-50 architecture					
All views	0.727 [0.708, 0.745]	0.714 [0.695, 0.735]	0.383	0.924 [0.913, 0.935]	0.918 [0.905, 0.930]	0.496
CC views	0.728 [0.701, 0.753]	0.713 [0.686, 0.741]	0.472	0.920 [0.902, 0.935]	0.924 [0.908, 0.940]	0.751
MLO views	0.726 [0.698, 0.751]	0.717 [0.691, 0.744]	0.642	0.927 [0.911, 0.942]	0.913 [0.895, 0.928]	0.240
	EfficientNet-B0 architecture					
All views	0.703 [0.683, 0.723]	0.699 [0.679, 0.718]	0.769	0.922 [0.912, 0.933]	0.914 [0.902, 0.925]	0.325
CC views	0.706 [0.678, 0.731]	0.676 [0.648, 0.704]	0.146	0.913 [0.897, 0.929]	0.907 [0.889, 0.924]	0.602
MLO views	0.701 [0.674, 0.728]	0.722 [0.695, 0.748]	0.308	0.932 [0.918, 0.947]	0.922 [0.906, 0.936]	0.368
	DenseNet-121 architecture					
All views	0.732 [0.714, 0.750]	0.711 [0.689, 0.730]	0.133	0.930 [0.918, 0.939]	0.912 [0.901, 0.924]	0.037
CC views	0.733 [0.706, 0.758]	0.716 [0.689, 0.745]	0.412	0.930 [0.914, 0.945]	0.917 [0.900, 0.934]	0.029
MLO views	0.731 [0.704, 0.757]	0.706 [0.678, 0.733]	0.206	0.929 [0.914, 0.944]	0.908 [0.891, 0.925]	0.009

Breast density classification accuracy on the test set. Numbers in parentheses are 95% CIs.

$p_{1}$: p-value for the difference in accuracy between DM and SM using a permutation test.

## Data Availability

The datasets analyzed during the current study are available from the corresponding author upon reasonable request. The codes used in this study are available in the GitHub repository at https://github.com/BIC-Lab/DL_BreastDensity_DM_SM.
